# Comparative study on the bioavailability and bioequivalence of rifapentine capsules in humans

**DOI:** 10.3389/fphar.2024.1463575

**Published:** 2025-01-17

**Authors:** Ying Qi, Pengfei Zhao

**Affiliations:** ^1^ Department of Radiology, Shengjing Hospital of China Medical University, Shenyang, China; ^2^ Department of Pharmacology, School of Pharmacy, China Medical University, Shenyang, China

**Keywords:** rifapentine, UPLC, bioavailability, bioequivalence, pharmacokinetics

## Abstract

**Introduction:**

: Rifapentine, a potent semi-synthetic member of the rifamycin class, is approved for the treatment of tuberculosis due to its effective bactericidal properties. It is essential to assess the bioequivalence and bioavailability of different rifapentine formulations to ensure consistent clinical outcomes. This study compares the pharmacokinetic profiles of test and reference rifapentine capsules in healthy male volunteers.

**Methods:**

In this single-dose, randomized, crossover study, 19 healthy male volunteers aged 18–40 received 0.6 g of either the test or reference rifapentine capsules. The reference is an NMPA-approved product, while the test is a modified version intended to match it in safety and efficacy; both contain the same active ingredient but may differ in excipients or manufacturing processes. Blood samples were collected at predefined intervals over a 84-h period following administration to measure rifapentine plasma concentrations using UPLC. Key pharmacokinetic parameters, including maximum concentration (C_max_), time to maximum concentration (T_max_), and area under the concentration-time curve (AUC), were calculated and analyzed for bioequivalence.

**Results:**

The pharmacokinetic analysis demonstrated that both formulations of rifapentine had similar absorption rates and extent of exposure. The mean Cmax, Tmax, and AUC values were closely aligned between the two formulations. Statistical analysis, including ANOVA and bioequivalence testing, confirmed that the 90% confidence intervals for the primary pharmacokinetic parameters (C_max_, AUC_0-t_, and AUC_0- ∞ _) fell within the acceptable range of 80%–125% for bioequivalence. Both formulations were well-tolerated with no serious adverse events reported.

**Discussion:**

The results of this study confirm the bioequivalence of the test and reference formulations of rifapentine under the conditions tested. These findings support the interchangeable use of these formulations in clinical practice for the treatment of tuberculosis. This study contributes to the body of evidence needed to ensure that patients receive a consistent therapeutic effect when administered either formulation of rifapentine.

**Conclusion:**

The bioequivalence demonstrated between the test and reference rifapentine capsules supports their use in clinical settings where rifapentine is indicated for tuberculosis therapy. This study provides a robust foundation for the regulatory approval of generic formulations of rifapentine, ensuring that patients have access to effective and lower-cost medication options.

## Highlights

• The study successfully demonstrated the bioequivalence of the test and reference formulations of rifapentine capsules, with pharmacokinetic parameters like C_max_, T_max_, and AUC being statistically comparable within the accepted bioequivalence range of 80%–125%.
• Utilizing advanced UPLC techniques, the study provided detailed pharmacokinetic analysis, ensuring accurate measurement of rifapentine concentrations in plasma over a defined period, thus reinforcing the reliability of the findings.
• Both rifapentine formulations were well-tolerated by the healthy male volunteers, with no serious adverse events reported, highlighting their safety for further clinical use.
• The confirmation of bioequivalence between the test and reference formulations supports their interchangeable use in treating tuberculosis, offering potential for cost reductions in treatment without compromising therapeutic efficacy.

## Introduction

**FIGURE 1:**
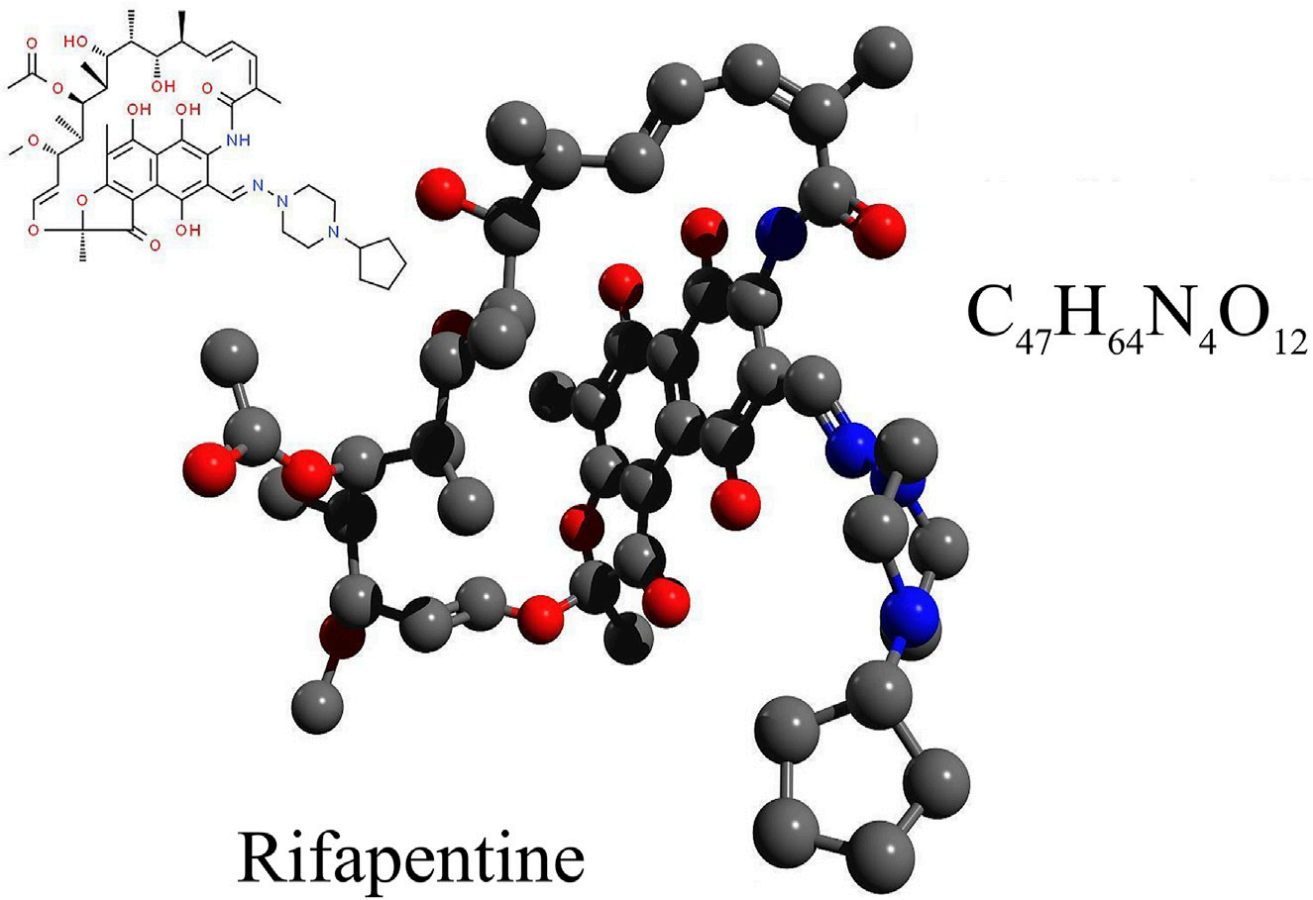
The chemical formula, structural formula, and spatial structure diagram of Rifapentine.

Rifapentine is a semi-synthetic, broad-spectrum bactericidal agent, with the molecular formula C_47_H_64_N_4_O_12_ and a molecular weight of 877.04, please refer to Figure 1. Its chemical designation is 3-[4-cyclopentyl-1-piperazinyl-iminomethyl]-rifamycin SV (Temple and Nahata, 1999). Rifapentine has been in clinical use in China since it was included in the 1996 edition of the Chinese National Essential Medicine List (Zheng et al., 2017). The U.S. Food and Drug Administration (FDA) (2001) approved rifapentine for the treatment of tuberculosis in 1998 (Roehr, 1998). As a derivative of rifamycin B, rifapentine belongs to the rifamycin family of antibiotics. It appears as a brick-red or dark red crystalline powder that is both odorless and tasteless. The compound dissolves easily in methanol and chloroform, has limited solubility in ethanol and acetone, and is almost completely insoluble in water and ether (Jenkin, 2017).

*In vitro* studies reveal that rifapentine possesses significant antimicrobial efficacy against *M. tuberculosis*, with a minimum inhibitory concentration (MIC) ranging from 0.03 to 0.25 mg/L. This makes it 2 to 10 times more potent than Rifampin (Rastogi et al., 2000; Bemer-Melchior et al., 2000; Mor et al., 1995; Arioli et al., 1981). Clinically, a bi-weekly treatment regimen with rifapentine achieves results comparable to daily Rifampin therapy, but with fewer side effects (Van den Boogaard et al., 2009). Follow-up studies 3 years post-treatment have shown a bacteriological relapse rate of 2.6% with rifapentine (Jarvis and Lamb, 1998), demonstrating its substantial long-term effectiveness in treating pulmonary tuberculosis with a relatively low recurrence rate. Moreover, rifapentine exhibits strong antibacterial activity against most Gram-positive bacteria, although its effectiveness against Gram-negative bacteria is weaker (Wu et al., 2015; Albano et al., 2021). When combined with isoniazid, rifapentine’s suppressive effects on *Mycobacterium tuberculosis* significantly exceed those observed with the sole use of Rifampin and isoniazid (Sterling et al., 2011). Additionally, rifapentine has been shown to prevent tuberculosis in HIV-positive individuals (Swindells et al., 2019).

Tuberculosis is an infectious disease caused by the *M. tuberculosis* complex, primarily affecting various organs throughout the body, with the lungs being the most common site (Daniel, 2006). This disease is one of the leading infectious diseases worldwide, with extremely high incidence and mortality rates, claiming over one million lives each year (Glaziou et al., 2018). According to the World Health Organization’s (WHO) 2022 Global Tuberculosis Report, there were 10.6 million new cases of tuberculosis in 2021, with an estimated 1.6 million deaths (Daley, 2019). Tuberculosis remains one of the significant epidemics among the top ten causes of death globally. Thus, the pharmacokinetics of drugs related to the treatment of tuberculosis will continue to be a focus of pharmacological research for an extended period.

Antituberculosis medications are broadly divided into first-line and second-line drugs. First-line agents include isoniazid, Rifampin, pyrazinamide, ethambutol, rifabutin, and rifapentine, each possessing a distinct mechanism of action. Notably, rifapentine’s pharmacological action is characterized by its binding to the subunit of DNA-dependent RNA polymerase, which inhibits bacterial RNA synthesis, halts the RNA transcription process, and ceases the synthesis of DNA and proteins, while having no impact on RNA polymerase in human and animal cells (Brodolin, 2014). Animal studies have demonstrated that this drug exhibits certain hepatotoxic effects and may have teratogenic impacts on fetuses (Jenkin, 2017). Recently, rifapentine has garnered considerable attention in clinical settings due to its pronounced antimicrobial properties and relatively low resistance rates (Rosenthal et al., 2007; Daniel et al., 2000; Conte et al., 2000).

Research indicates substantial interindividual variability in the bioavailability of rifapentine during administration (Francis et al., 2019; Savic et al., 2014; Weiner et al., 2004). The absorption of the drug in the gastrointestinal tract is slow and incomplete, influenced significantly by the presence of food. For example, bioavailability increases by 55% when a 600 mg tablet is taken with food, compared to fasting, with corresponding increases of 44% and 43% in peak concentration (C_max_) and area under the curve (AUC_0- ∞ _), respectively (Keung et al., 1995). Volunteer studies have also recorded the time to reach peak concentration (T_max_) as ranging between 4.8 and 6.6 h (Jarvis and Lamb, 1998; Temple and Nahata, 1999). The drug’s protein binding rate exceeds 98% (Keung et al., 1998), and its oral half-life ranges from 14 to 16 h (Egelund et al., 2014). *In vivo*, rifapentine is predominantly distributed in the liver, with secondary distribution in the kidneys and high concentrations in other tissues, though it poorly penetrates the blood-brain barrier (Zurlinden et al., 2016). In the liver, rifapentine undergoes deacetylation by esterase to form 25-desacetyl rifapentine. This metabolite deacetylates more slowly than Rifampin, significantly reducing protein binding and resulting in the formation of inactive 3-formylrifamycin upon hydrolysis (Langdon et al., 2005). The drug and its metabolites primarily undergo hepatic-intestinal recycling; some are excreted into the intestine through bile, where they can be reabsorbed and then expelled with the feces, with only a minor portion eliminated *via* urine (Reith et al., 1998). Moreover, as rifapentine is a hepatic enzyme inducer, it accelerates the metabolism of itself and several other medications, requiring careful monitoring of drug interactions during clinical combination therapies and home medication management (Ling et al., 2023; Zheng et al., 2017).

In conclusion, rifapentine is a crucial antituberculosis drug, making the study of its pharmacokinetics in the human body especially important. Given the limited public data on its bioequivalence studies, this research conducted a single-dose cross-over oral trial with rifapentine capsules in healthy adult males, using both the test and reference formulations. By estimating the pharmacokinetic parameters and assessing bioavailability, this study evaluates the bioequivalence of the test drug to the reference, aiming to provide cost-effective, high-quality options for patient treatment. Additionally, this project has developed a rapid method to detect rifamycin-class drugs in human plasma, offering a benchmark for the rapid testing of similar medications.

## Materials and methods

### Formulations and subject selection

The test formulation of rifapentine capsules was provided by Shenyang Everbright Pharma Co., Ltd. Each size 0 gelatin capsule contains 0.15 g of rifapentine (lot no. 20190506, with a 2-year shelf life). The reference formulation, produced by Changzheng Pharmaceutical Co., Ltd., is a commercially available, NMPA-approved product widely used for tuberculosis treatment. Each size 0 gelatin capsule of the reference formulation also contains 0.15 g of rifapentine (lot no. 20190511, with a 2-year shelf life). This study comprised 19 healthy male volunteers, all of East Asian descent and Han ethnicity, aged between 18 and 40 years, with a Body Mass Index (BMI) ranging from 19 to 25. All volunteers had normal results in routine blood and urine tests, liver and kidney function assessments, coagulation profiles, electrocardiograms, and chest X-rays. Adhering to the Declaration of Helsinki and Good Clinical Practice (GCP) guidelines, written informed consent was secured from all participants before any screening or other study-related activities commenced. Participants were fully informed about the drug’s characteristics, the study’s objectives, the associated risks, and their rights and obligations before consenting. Researchers meticulously documented all data on Case Report Forms (CRFs) based on the initial assessments of the participants. Monitors ensured that all CRFs were filled out correctly and comprehensively, according to the study protocol. Any amendments were made clearly, with the researcher’s signature and date. After review and approval by monitors, the trial’s typical spectra, plasma drug concentration data, and CRFs were submitted for statistical analysis by clinical data analysts. The study was conducted at the National Institute for Drug Clinical Experiments, affiliated with the First Hospital of China Medical University.

## Study design

### Route of administration and dosage design

Participants were instructed to fast overnight for more than 10 h. The following morning at 7:00 a.m., they administered the prescribed medication on an empty stomach, accompanied by 250 mL of warm water. Each capsule contained 0.15 g of the formulation. The dosage was established at 0.6 g per dose (4 capsules), based on the package insert of the reference formulation and a thorough review of clinical study data for the test formulation (Jarvis and Lamb, 1998; Dooley et al., 2008; Weiner et al., 2004; Bock et al., 2002).

### Design of the medication protocol

The study was designed as a single-center, open-label, randomized trial. Participants were divided into two groups, A and B, with 10 individuals in each group. A two-period crossover design was used, with specific dosing protocols detailed in Supplementary Table S1. Participants were instructed not to consume alcohol, caffeinated beverages, or juice the day before and during the trial. After fasting for 10 h, participants took their assigned medication on an empty stomach the following morning. Group A took four capsules of the test formulation of rifapentine, and Group B took four capsules of the reference formulation of rifapentine, each with 250 mL of warm water. Participants were not allowed to drink water within the first 2 h after dosing and were provided a standardized meal (low-fat diet) 4 h post-dose. After a 1-week washout period, the groups switched medications. Each participant in the same group took their medication at 2-min intervals, and blood samples were also drawn at 2-min intervals. Blood samples were protected from light, placed in an ice bath, and centrifuged quickly for plasma separation, then stored at −70°C. Based on the results of a preliminary trial, 4 mL of blood was drawn from the antecubital vein into a heparinized tube, centrifuged at 4°C at 4,000 r/min for 10 min, and the plasma was then stored at −70°C for future analysis. The washout period lasted for 7 days, with the second cycle of medication administration and blood sampling mirroring the first cycle.

### Pre-trial

Before the trial, researchers should inquire about the participants’ medical history and any allergies to medications, followed by a physical examination and laboratory tests. Participants are required to fast after 7 p.m. the night before each trial day and take the medication on an empty stomach the following morning, accompanied by a standardized low-fat meal. During the trial, strenuous activities, smoking, alcohol consumption, and the intake of caffeinated beverages are prohibited. No non-trial medications are allowed.

### Trial day

The trial utilizes a two-period, two-formulation crossover design to mitigate the effects of cycle variation and individual differences on the outcomes. Twenty healthy participants are randomly divided into two groups of ten. After fasting for more than 10 h, participants in the control group take four capsules of the reference formulation, and those in the test group take four capsules of the test formulation, both with 250 mL of warm water. Blood samples (4 mL each) are collected at pre-dose (0 h) and at 1, 2, 3, 4, 5, 7, 9, 12, 24, 36, 48, 72, and 84 h post-dose, placed into heparinized tubes, and centrifuged at 4°C for 10 min at 4,000 rpm. The plasma is then stored at −70°C. Participants may drink water 2 h after dosing and consume a standardized low-fat meal 4 h later. Blood draws are conducted in a clinical monitoring room. In case of adverse reactions, emergency measures should be taken, and the trial may be stopped if necessary. After a 7-day washout period, the groups cross over and repeat the procedure. Participants should avoid strenuous activities and prolonged bed rest during the trial. They stay in the observation room for 12 h post-dosing under clinical supervision to monitor any adverse reactions and overall condition. Emergency interventions are prepared for severe reactions, and all incidents are duly recorded. The monitored adverse reactions included allergic reactions (such as skin itching, redness, swelling, rash, wheezing, chest tightness, difficulty breathing, and difficulty swallowing or speaking), gastrointestinal issues (including diarrhea, abdominal pain, nausea, vomiting, and loss of appetite), central nervous system effects (such as abnormal thoughts and behaviors), cardiovascular symptoms (including abnormal heart rate and chest pain), specific reactions (such as swelling of the eyes, face, lips, tongue, throat, or limbs), flu-like symptoms (such as fever, chills, muscle soreness, fatigue, and headache), liver dysfunction (indicated by dark urine or yellowing of the skin or eyes), musculoskeletal reactions (such as joint pain or swelling), and blood system reactions (such as hemolytic anemia and thrombocytopenic purpura).

### Post-trial examination

On the first day after the end of the second trial period, follow-up laboratory tests are conducted. Any clinically significant abnormalities are tracked until they return to normal.

### Chemical materials

Rifapentine reference standard (99.6%, National Institutes for Food and Drug Control, China), Rifampin reference standard (99.7%, National Institutes for Food and Drug Control, China); methanol and acetonitrile provided by Thermo Fisher Scientific, chromatography grade reagents; ultrapure water from Millipore (Bedford, MA, United States); blank plasma supplied by Shengjing Hospital.

### Instrumentation and conditions

ACQUITY™ UPLC system (Waters Corporation, Milford, MA, United States), which includes a quaternary high-pressure pump system, online degassing system, autosampler, column heater, and TUV detector. Data acquisition and processing were conducted using the Empower Chromatography Workstation; Milli-Q Gradient A10 Ultrapure Water System (Millipore Inc., United States); Tianmei D-2000 Chromatography Data Workstation Software, produced by Tianmei Technology Co., Ltd.; AT-330 Column Heater, manufactured by Autoscience Instruments Co., Ltd. in Tianjin; TGL-16C Centrifuge, made by Feige Instrument Co., Ltd.; XW-80A Mini Vortex Mixer (Shanghai Huaxi Instrument Factory); and XS105 Mettler Electronic Balance (Shanghai Mettler-Toledo Instruments Co., Ltd.). Chromatographic conditions: the column used was a Dima C8, 250 mm × 4.6 mm (Diamond column), column temperature: 35°C, mobile phase: methanol: water = 76:24, flow rate: 1.2 mL/min, injection volume: 50 μL, absorbance range: 0.25 AU, detection wavelength: 340 nm.

### Plasma sample processing

Accurately transfer 100 μL of plasma into a 1.5 mL centrifuge tube. Precisely add 10 μL of a Rifampin internal standard solution (622 μg/mL) and vortex for 30 s. Subsequently, accurately add 190 μL of acetonitrile, vortex for an additional 2 min, then centrifuge at 14,000 rpm for 10 min. Carefully collect the supernatant, inject 50 μL into the analytical system. Ensure all procedures are conducted away from light to protect the samples.

### Method specificity

**FIGURE 2:**
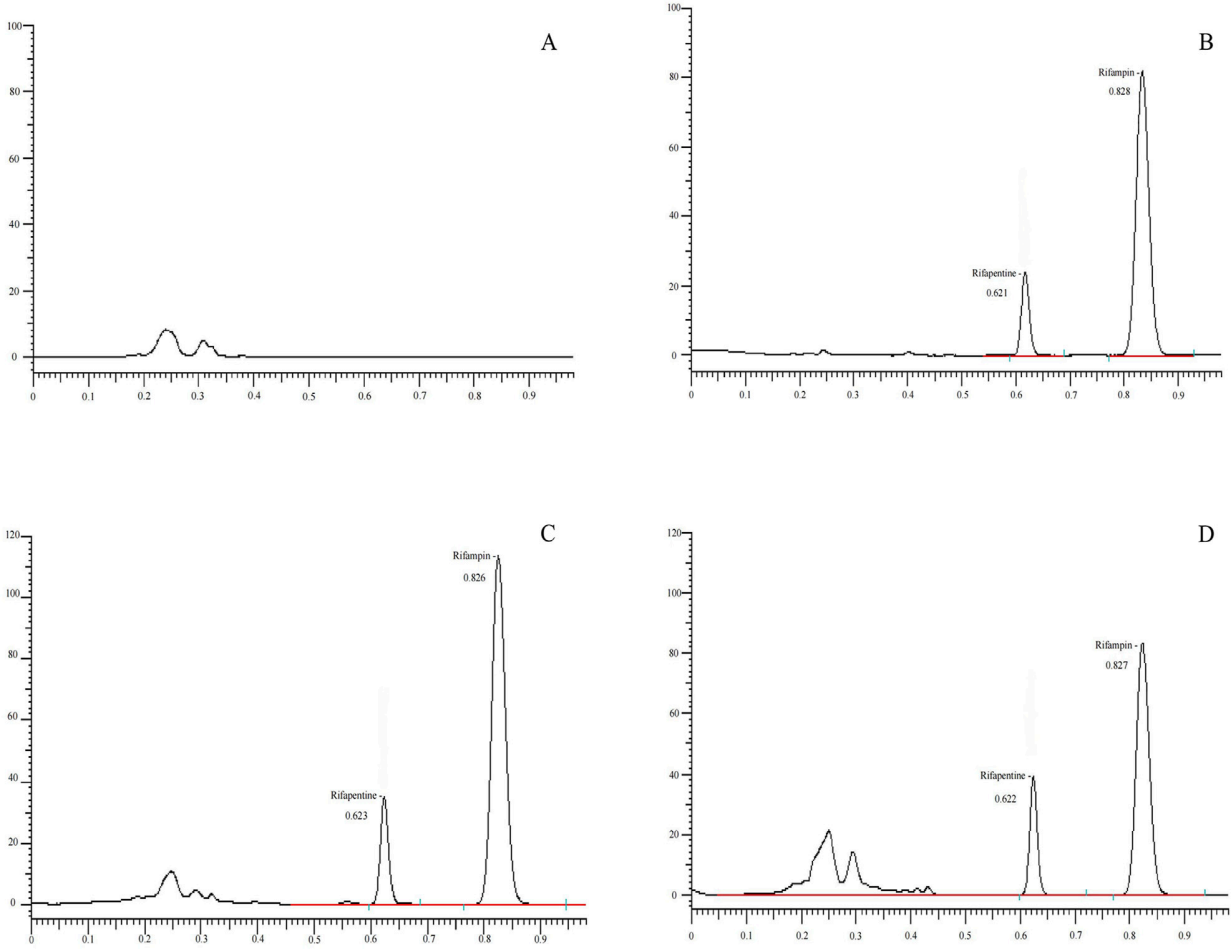
UPLC chromatogram of Rifapentine in plasma. **(A)** Chromatogram of the blank plasma sample. **(B)** Chromatogram of rifapentine standard (16 μg/mL) and I.S. rifampin standard (62.2 μg/mL). **(C)** Chromatogram after adding rifapentine (16 μg/mL) and rifampin standard (62.2 μg/mL) to blank plasma. **(D)** Chromatogram of rifapentine and rifampin in plasma from subject No. 1, 4 h after oral administration of rifapentine capsules.

Accurately dispense 100 μL of mixed blank plasma from six different sources and process following the 'Plasma Sample Processing Method,’ beginning with 'vortex for 30 s,’ to produce Figure 2A. Introduce 10 μL of a 160 μg/mL rifapentine standard solution and 10 μL of the internal standard solution, both diluted with 100 μL acetonitrile, to generate Figure 2B. Add rifapentine standard solution to the blank plasma to achieve a 16 μg/mL drug-containing plasma concentration, and process as per the 'Plasma Sample Processing Method’ to obtain Figure 2C. Collect plasma samples from Subject No. 1, 4 hours post-drug administration, and follow the 'Plasma Sample Processing Method’ to produce Figure 2D. Rifapentine’s retention time is approximately 0.82 min, and the internal standard, rifampin, is around 0.62 min. The results confirm that endogenous substances in the plasma do not interfere with the quantification of rifapentine and the internal standard.

### Calibration curve and LLOQ

Accurately dispense 10 μL of rifapentine standard solutions at various concentrations into seven blank centrifuge tubes, and add 100 μL of blank plasma to create plasma samples containing rifapentine at concentrations of 0.05, 0.25, 0.50, 1.00, 5.00, 10.00, and 20.00 mg per liter. Proceed according to the 'Plasma Sample Processing Method,’ starting from the step where 10 μL of the rifampin internal standard solution (622 μg/mL) is precisely added. Analyze the samples to establish a standard curve for rifapentine. The concentration of the test substance (X, μg/mL) is plotted on the *x*-axis, and the ratio of the peak area of the test substance (As) to that of the internal standard (Ai) (As/Ai) is plotted on the *y*-axis, represented as Y. Linear regression is performed using a weighting factor (weight coefficient: 1/X^2^) to obtain the linear equation for the standard curve. The average standard curve results, validated over 3 days, are presented in Supplementary Tables S2–S4, and Supplementary Figure S1, which includes three standard curves per day. The findings confirm that rifapentine maintains a robust linear relationship within the concentration range of 0.05–20.00 μg/mL, encompassing the plasma concentrations of rifapentine in humans, with a quantitation limit of 0.05 μg/mL (S/N > 3).

Take 100 μL of blank plasma and add 10 μL of a 0.5 μg/mL rifapentine standard solution to prepare a sample with a final rifapentine concentration of 0.05 μg/mL in the plasma. On the second day of method validation, analyze six samples and calculate the concentration of each sample using the day’s standard curve. The data are presented in Supplementary Table S5. The results demonstrate that the UPLC-UV method can quantify rifapentine in plasma with a lower limit of quantification (LLOQ) of 0.05 μg/mL.

### Precision and accuracy

Dispense 10 μL of rifapentine standard solution at varying concentrations into separate blank centrifuge tubes, then add 100 μL of blank plasma to prepare samples with drug concentrations of 0.1, 2.5, and 16 μg/mL, representing low, medium, and high concentration levels in the plasma. Follow the 'Plasma Sample Processing Method’ for preparation. Conduct this preparation and analysis across three analytical batches on different days, with each batch containing low, medium, and high concentration levels, analyzing six samples per concentration. Calculate the intra-day and inter-day relative standard deviation (RSD), which was found to be less than 10%. The results are documented in Supplementary Table S6.

### Extraction recovery

Begin by adding 100 μL of blank plasma into each empty centrifuge tube, followed by the precise addition of 10 μL of rifapentine standard solution at various concentrations. Proceed according to the 'Standard Curve and Lower Limit Quantitation Plasma Sample Handling Method’ to prepare samples at low, medium, and high concentrations. Conduct three analyses per concentration, determining the respective drug peak area, As(H), and the internal standard peak area, Ai(H). Subsequently, mix 10 μL of rifapentine standard solution at varying concentrations with 10 μL of internal standard solution, dilute with acetonitrile, and perform an injection analysis to ascertain the corresponding drug peak area As(D) and internal standard peak area Ai(D). The extraction recovery rates are calculated as follows: for the drug, As(H)/As(D) × 100%; for the internal standard, Ai(H)/Ai(D) × 100%. These results are detailed in Supplementary Table S7, which shows that the extraction recovery rates for low, medium, and high concentrations range from 102.60% to 109.55%, and the internal standard recovery rates vary from 99.19% to 112.04%.

### Sample stability

Start by adding 10 μL of rifapentine standard solution at different concentrations into blank centrifuge tubes, followed by 100 μL of blank plasma to prepare plasma samples with drug concentrations of 0.10 and 16.00 μg/mL at low and high levels, respectively. Prepare five sets of these concentrations, with each set comprising three samples. For one set, follow the 'Plasma Sample Processing Method’ and analyze the samples after leaving them at room temperature for 0, 24, and 36 h to evaluate the stability of plasma samples under room temperature conditions. Also assess the stability of plasma samples after thawing for 8 h. Another set is to be frozen at −70°C and subjected to three freeze-thaw cycles, then processed and analyzed according to the 'Plasma Sample Processing Method’ to evaluate the stability after repeated freeze-thaw cycles. Additionally, freeze two more sets at −70°C and analyze them after 15 and 30 days, respectively, following the 'Plasma Sample Processing Method’ to assess the long-term stability under freezing conditions, with each set analyzed in triplicate. Concurrently, evaluate the stability of the rifapentine standard solution after remaining at room temperature for 8 h and after being frozen at −20°C for 30 days. The results, as shown in Supplementary Tables S8, S9, indicate an RSD of less than 10%, demonstrating good stability of rifapentine under room temperature, repeated freeze-thaw, and long-term freezing conditions.

### Accompanying standard curves and quality control in sample analysis

To reduce systematic errors, an accompanying standard curve is generated for each batch of sample analyses to calculate the concentrations of medication in the blood. This curve is constructed using a weighted regression method (W = 1/X^2^). Additionally, quality control samples at low, medium, and high concentrations—0.1, 2.5, and 16 μg/mL, respectively—are measured. The results can be found in Supplementary Tables S10, S11.

### Data processing

Pharmacokinetic parameters are calculated and statistically analyzed using DAS 2.1 and SPSS 21.0 software. The analysis primarily focuses on descriptive statistics, with inferential statistics serving as supplementary information. Quantitative data are presented as mean ± standard deviation (Mean ± SD). To determine whether changes in parameters before and after administration or between dosage groups are statistically significant, a p-value of less than 0.05 is considered indicative of a meaningful difference.

### Administration trial results expression and analysis methods

Utilize the drug concentration-time data (c-t) obtained from each subject in the trial to plot the c-t curves. Additionally, compile a table of mean drug concentrations and standard deviations at each time point, and construct a graph of the average plasma concentration curves, including standard deviations. Provide the T_max_, C_max_, AUC_0-t_, AUC_0- ∞ _, and T_1/2_ for each participant who received the test and reference formulations. Tmax and Cmax should be reported as observed values, while the AUC should be calculated using the trapezoidal rule.

$$T_{1/2}=\frac{0.693}{\lambda_{z}}$$ (1)

$$\begin{array}{l} {\text{AUC}}_{0-t_{n}}=\sum \frac{t_{i+1}-t_{i}}{2}\left(C_{i+1}+C_{i}\right)\\ {\text{AUC}}_{0-\infty }=\sum \frac{t_{i+1}-t_{i}}{2}\left(C_{i+1}+C_{i}\right)+\frac{C_{t}}{\lambda_{z}} \end{array}$$ (2)

The terminal elimination constant, λ_z_, is calculated from the slope of the linear section of the logarithmic plasma concentration-time curve towards the end. C_t_ denotes the concentration of the drug in the plasma at the final sampling point.

$$F=\frac{{\text{AUC}}_{\left(T\right)}}{{\text{AUC}}_{\left(R\right)}}\times 100\%$$ (3)

Statistical Analysis: For variance analysis, after log transformation of C_max_, AUC_0-t_, and AUC_0- ∞ _, an analysis of variance (ANOVA) for a crossover design is used to assess the statistical significance of variations between formulations, individuals, and periods. For bioequivalence testing, C_max_, AUC_0-t_, and AUC_0- ∞_ are subjected to two-sided, one-sample T-tests after log transformation. Equivalence is established if the AUC for the reference formulation falls within 80%–125%, and C_max_ within 70%–143% of the corresponding parameters.

① Test Hypothesis:

$$H_{0}:\eta_{T}-\eta_{R}\leq {\text{lnr}}_{1}\mathrm{or}n_{T}-\eta_{R}\geq \ln r_{2}$$

$$H_{1}:{\text{lnr}}_{1}<\eta_{T}-\eta_{R}\;<\ln r_{2}$$

η_T_ and η_R_ represent the mean parameter data for the test formulation and reference formulation.

② Calculate the Statistic:

$$T_{1}=\frac{\left(\eta_{T}-\eta_{R}\right)-{\text{lnr}}_{1}}{S\cdot \sqrt{2/n}}$$

$$T_{2}=\frac{{\text{lnr}}_{2}-\left(\eta_{T}-\eta_{R}\right)}{S\cdot \sqrt{2/n}}$$

S represents the square root of the mean square error derived from the analysis of variance. T_1_ and T_2_ follow a T-distribution with ν degrees of freedom, which is denoted as T_1-α_(ν). If both T_1_ >T_1-α_(ν) and T_2_ >T_1-α_(ν) are true, then the null hypothesis H_0_ is rejected, and the alternative hypothesis H_1_ of bioequivalence is accepted. T_max_ is analyzed using non-parametric testing. C_max_, AUC_0-t_ and AUC_0- ∞_ are subjected to logarithmic transformation for Population Bioequivalence Testing, while T_max_ is not transformed; these results are provided for reference only.

$$\text{If }\sigma_{TR}^{2}>\sigma_{T0}^{2},\eta_{1}={\left(\mu_{T}-\mu_{R}\right)}^{2}+\left(\sigma_{\text{TT}}^{2}-\sigma_{\text{TR}}^{2}\right)-\theta_{P}\sigma_{\text{TR}}^{2}$$

$$\text{If }\sigma_{TR}^{2}\leq \sigma_{T0}^{2},\eta_{2}={\left(\mu_{T}-\mu_{R}\right)}^{2}+\left(\sigma_{\text{TT}}^{2}-\sigma_{\text{TR}}^{2}\right)-\theta_{P}\sigma_{T0}^{2}$$

If the upper limit of the 95% confidence interval for either η1 or η2 is less than zero, population bioequivalence is confirmed. The term $\sigma_{T0}^{2}$ is the total variance for a specific constant, and θ_p_ is the threshold value for establishing population bioequivalence. $\mu_{T}$ , $\mu_{R}$ represent the overall mean values of the parameters for the test and reference formulations, respectively, with their total variances denoted as $\sigma_{\text{TT}}^{2}$ and $\sigma_{TR}^{2}$ .

## Results

### Determination of plasma samples and analysis of results

**FIGURE 3:**
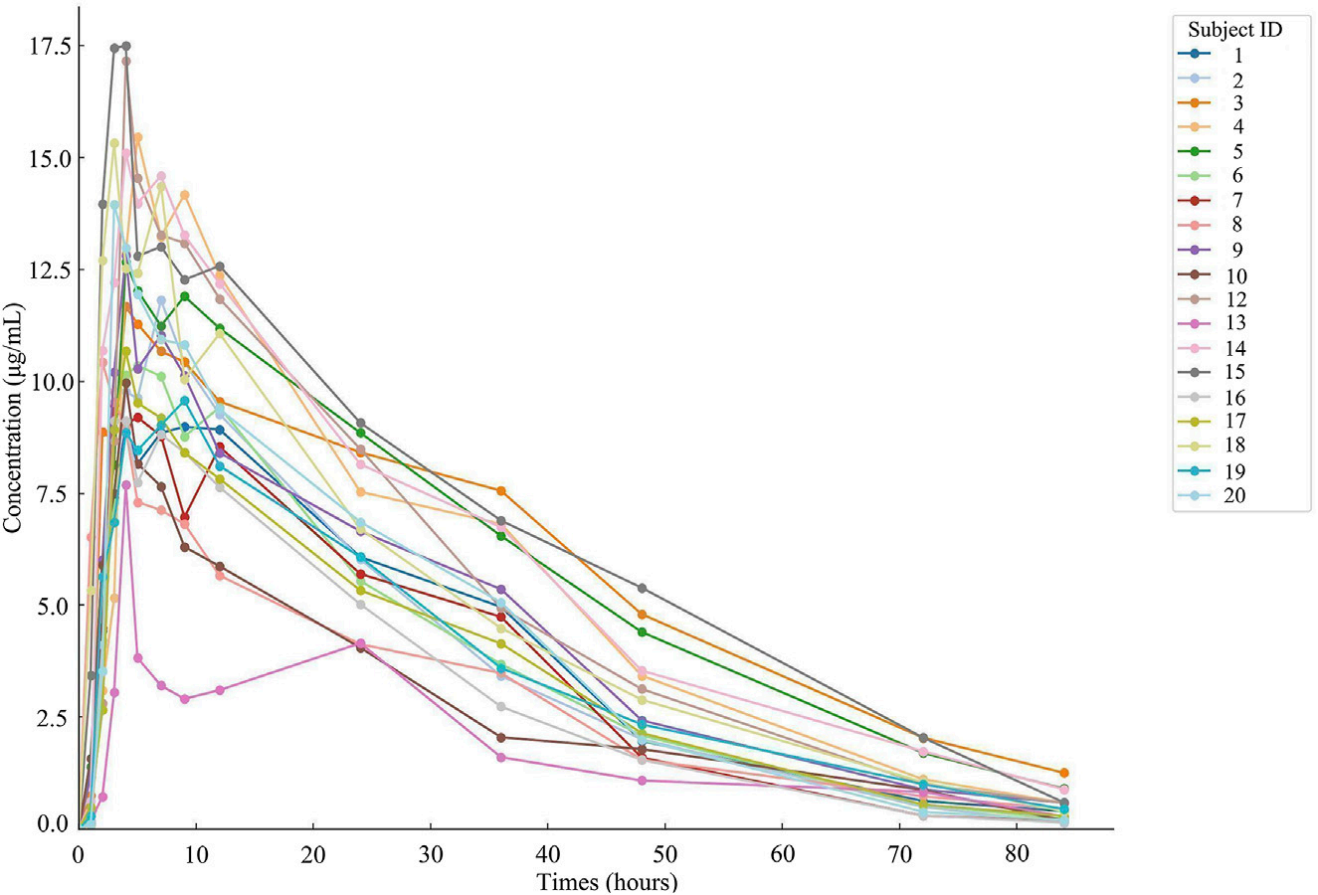
Plasma concentration-time curve of the drug after 19 subjects orally administered the reference formulation.

**FIGURE 4:**
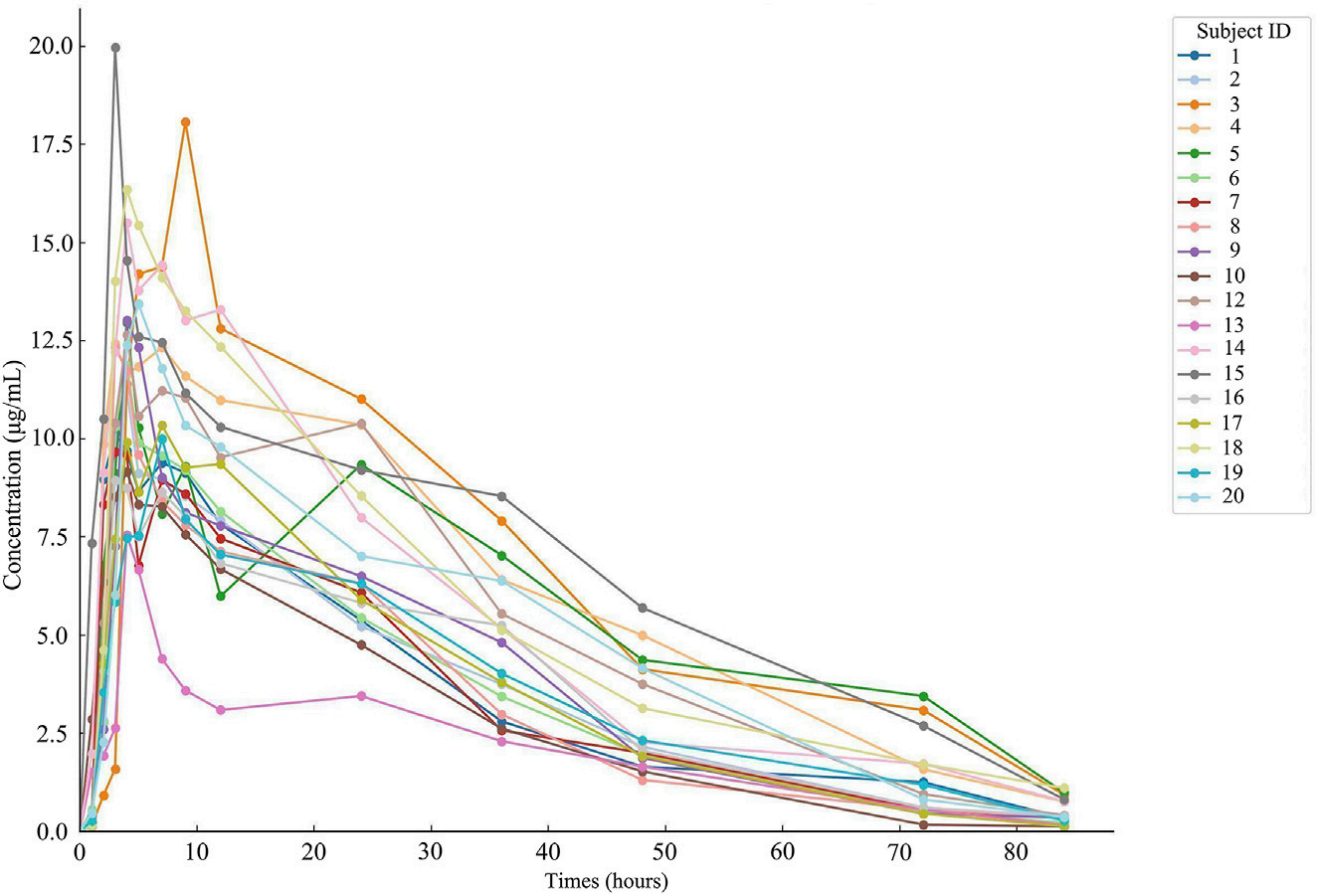
Plasma concentration-time curve of the drug after 19 subjects orally administered the test formulation.

**FIGURE 5:**
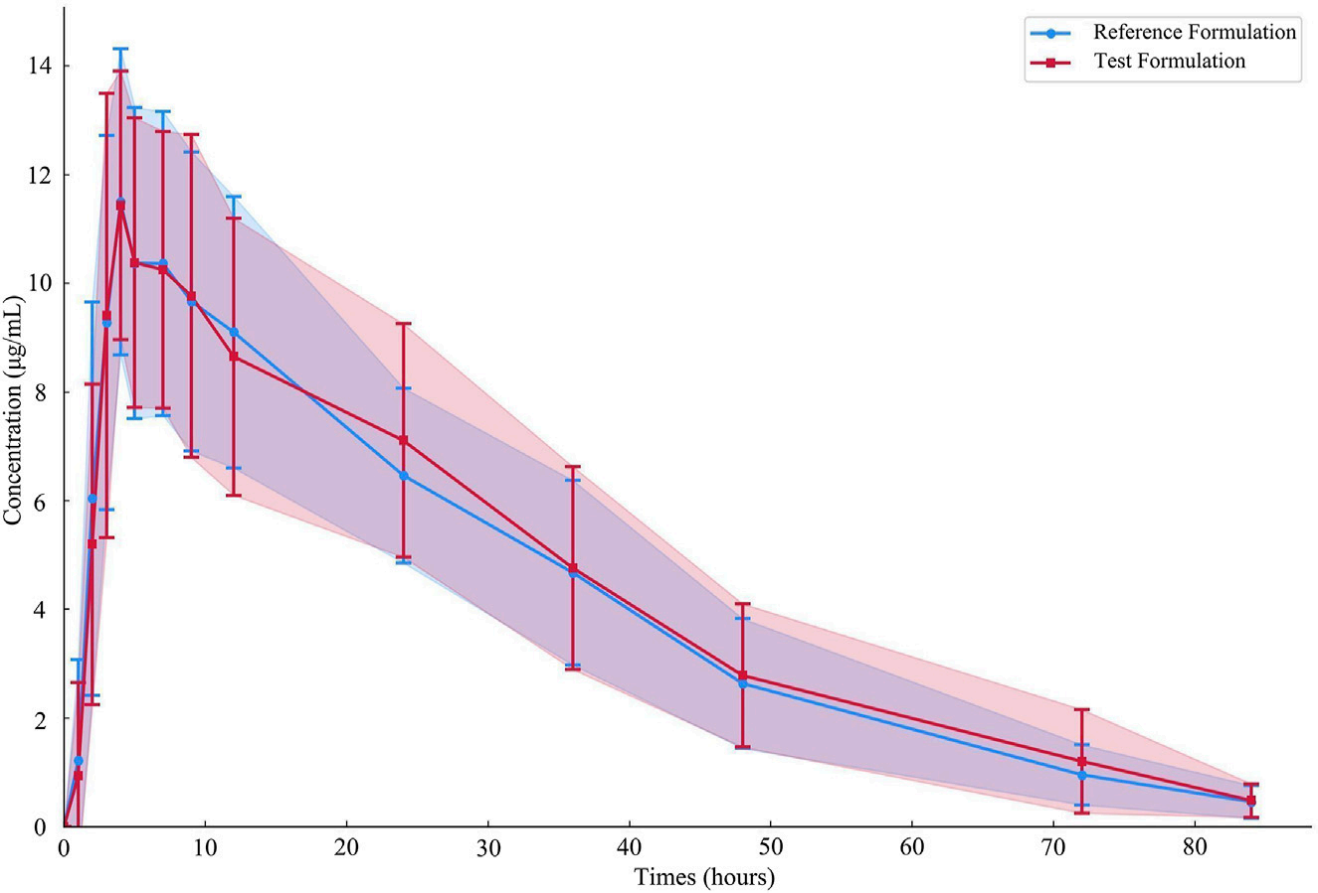
Average plasma concentration-time curves of the drug after 19 subjects orally administered either the test formulation or the reference formulation.

Nineteen participants orally administered 0.6 g of either the test or the reference formulation of rifapentine capsules. The rifapentine plasma concentration-time data were obtained using UPLC, as shown in Supplementary Tables S12, S13. Plasma concentration-time curves for the nineteen healthy participants who took the reference formulation can be found in Figure 3, while those for the test formulation are presented in Figure 4, and a comparison of the mean values is depicted in Figure 5.

### Pharmacokinetic calculations and bioequivalence

C_max_ and T_max_ are measured values. Other parameters are calculated using Equations 1, 2. The results are presented in Table 1 and 2. Relative bioavailability is calculated according to Equation 3. The ratio of AUC_0-84_, denoted as F, is used as the numeric value for relative bioavailability, and the ratio of AUC_0- ∞ _, denoted as F′, is used as a reference value. The results for F and F′ are shown in Table 3. The variance analysis results for ln AUC_0-84_, ln AUC_0- ∞ _, and ln C_max_ between the two formulations are presented in Tables 4–6. These results indicate no statistically significant differences between the formulations for ln AUC_0-84_, ln AUC_0- ∞ _, and ln C_max_; no statistically significant period differences for ln C_max_; however, significant period differences exist for ln AUC_0-84_ and ln AUC_0- ∞_ (P < 0.05); and highly significant inter-individual differences for ln AUC_0-84_, ln AUC_0- ∞ _, and ln C_max_ (P < 0.001). The results of the two-sided one-sample t-tests and the [1-2α]% confidence interval analysis for ln AUC_0-84_, ln AUC_0- ∞ _, and ln C_max_ are shown in Tables 7–9. The results in Tables 7, 8 indicate that the null hypothesis H_0_ for the pharmacokinetic parameters ln AUC_0-84_ and ln AUC_0- ∞_ of the two formulations is rejected in favor of the alternative hypothesis H_1_, and the results demonstrate that the [1-2α]% confidence intervals for ln AUC_0-84_ and ln AUC_0- ∞_ fall within the 80%–125% range, indicating bioequivalence in terms of absorption between the two formulations. Table 9 shows that the null hypothesis H_0_ for the pharmacokinetic parameter ln C_max_ is rejected in favor of H_1_, and the results indicate that the [1-2α]% confidence interval for ln C_max_ falls within the 70%–143% range, suggesting bioequivalence in peak concentration between the two formulations. The non-parametric test results for T_max_ are presented in Table 10.

**TABLE 1:** Pharmacokinetic parameters of the 0.6 g reference formulation of oral rifapentine capsules in 19 subjects.

Subject code	T_1/2_ (h)	C_max_ (μg/mL)	T_max_ (h)	AUC_0-t_ (ng/ml·h)	AUC_0- ∞ _ (μg/ml·h)	AUC_0-t/_AUC_0- ∞ _
1	14.616	8.995	4	321.900	329.373	0.977
2	13.182	11.815	7	314.286	319.311	0.984
3	18.546	11.679	4	488.294	521.842	0.936
4	13.588	15.458	5	456.435	468.032	0.975
5	18.347	12.671	4	482.430	511.342	0.943
6	12.892	10.357	5	309.860	315.212	0.983
7	11.901	9.468	3	296.356	299.470	0.990
8	16.078	10.435	2	255.167	265.345	0.962
9	16.441	12.816	4	363.242	375.836	0.966
10	13.710	9.972	4	234.502	238.236	0.984
12	15.499	17.158	4	437.770	450.709	0.971
13	18.823	7.692	4	159.525	167.126	0.955
14	18.683	15.112	4	492.391	516.614	0.953
15	21.857	17.497	4	551.555	596.212	0.925
16	11.769	9.120	3	257.155	259.646	0.990
17	12.473	10.672	4	298.951	304.146	0.983
18	17.024	15.325	3	408.910	424.826	0.963
19	15.899	9.572	9	316.029	326.213	0.969
20	11.698	13.953	3	354.721	358.005	0.991
Mean	15.422	12.093	4.211	357.867	370.921	0.968
SD	2.914	2.921	1.548	104.519	114.284	0.019
RSD (%)	18.9	24.2	36.8	29.2	30.8	2.0

**TABLE 2:** Pharmacokinetic parameters of the 0.6 g test formulation of oral rifapentine capsules in 19 subjects.

Subject code	T_1/2_ (h)	C_max_ (μg/mL)	T_max_ (h)	AUC_0-t_ (ng/ml·h)	AUC_0- ∞ _ (μg/ml·h)	AUC_0-t/_AUC_0- ∞ _
1	15.724	10.144	3	294.689	302.345	0.975
2	12.891	11.232	4	295.996	301.815	0.981
3	16.858	18.080	9	568.136	590.922	0.961
4	17.737	12.428	3	512.122	537.414	0.953
5	16.881	12.956	4	471.853	495.891	0.952
6	12.967	11.864	4	295.576	300.655	0.983
7	14.870	9.680	3	285.208	293.187	0.973
8	19.894	12.328	3	279.842	290.522	0.963
9	14.317	13.018	4	318.228	325.358	0.978
10	11.196	9.162	4	244.754	246.751	0.992
12	12.801	12.642	4	444.498	452.020	0.983
13	17.993	7.544	4	174.050	183.135	0.950
14	17.632	15.499	4	454.903	474.445	0.959
15	21.669	19.965	3	570.170	627.184	0.909
16	15.225	8.935	3	304.709	313.200	0.973
17	11.682	10.347	7	308.540	312.291	0.988
18	20.424	16.351	4	470.284	503.264	0.934
19	20.015	10.002	7	309.857	332.320	0.932
20	10.278	13.439	5	416.532	421.947	0.987
Mean	15.845	12.401	4.316	369.471	384.456	0.965
SD	3.350	3.221	1.635	114.334	124.561	0.022
RSD (%)	21.1	26.0	37.9	30.9	32.4	2.3

**TABLE 3:** Relative bioavailability and C_max_ percentages (C_T_/C_R_) for test formulation (T) and reference formulation (R).

Subject code	C_max_ (μg/mL)			AUC_0-t_ (μg/ml·h)			AUC_0- ∞ _ (μg/ml·h)		
	T	R	T/R	T	R	F (%)	T	R	F (%)
1	10.1	9.0	1.1	294.7	321.9	91.5	302.3	329.4	91.8
2	11.2	11.8	1.0	296.0	314.3	94.2	301.8	319.3	94.5
3	18.1	11.7	1.5	568.1	488.3	116.4	590.9	521.8	113.2
4	12.4	15.5	0.8	512.1	456.4	112.2	537.4	468.0	114.8
5	13.0	12.7	1.0	471.9	482.4	97.8	495.9	511.3	97.0
6	11.9	10.4	1.1	295.6	309.9	95.4	300.7	315.2	95.4
7	9.7	9.5	1.0	285.2	296.4	96.2	293.2	299.5	97.9
8	12.3	10.4	1.2	279.8	255.2	109.7	290.5	265.3	109.5
9	13.0	12.8	1.0	318.2	363.2	87.6	325.4	375.8	86.6
10	9.2	10.0	0.9	244.8	234.5	104.4	246.8	238.2	103.6
12	12.6	17.2	0.7	444.5	437.8	101.5	452.0	450.7	100.3
13	7.5	7.7	1.0	174.0	159.5	109.1	183.1	167.1	109.6
14	15.5	15.1	1.0	454.9	492.4	92.4	474.4	516.6	91.8
15	20.0	17.5	1.1	570.2	551.6	103.4	627.2	596.2	105.2
16	8.9	9.1	1.0	304.7	257.2	118.5	313.2	259.6	120.6
17	10.3	10.7	1.0	308.5	299.0	103.2	312.3	304.1	102.7
18	16.4	15.3	1.1	470.3	408.9	115.0	503.3	424.8	118.5
19	10.0	9.6	1.0	309.9	316.0	98.0	332.3	326.2	101.9
20	13.4	14.0	1.0	416.5	354.7	117.4	421.9	358.0	117.9
Mean	12.4	12.1	1.0	369.5	357.9	103.4	384.5	370.9	103.8
SD	3.2	2.9	0.2	114.3	104.5	9.6	124.6	114.3	10.0
RSD (%)	26.0	24.2	16.1	30.9	29.2	9.3	32.4	30.8	9.7

**TABLE 4:** Analysis of Variance Results for lnAUC_0-84_ Between Two Formulations.

Source of variation	SS	DF	MS	F	P
Total Variation	3.554	37	0.096		
Inter-formulation Variation	0.008	1	0.008	1.965	0.179
Inter-period Variation	0.008	1	0.008	1.930	0.183
Inter-subject Variation	3.469	18	0.193	47.279	0.000
Total Residuals	0.069	17	0.004		

**TABLE 5:** Analysis of Variance Results for lnAUC_0- ∞_ Across Two Formulations.

Source of variation	SS	DF	MS	F	P
Total Variation	3.786	37	0.102		
Inter-formulation Variation	0.010	1	0.010	2.343	0.144
Inter-period Variation	0.009	1	0.009	1.921	0.184
Inter-subject Variation	3.691	18	0.205	46.131	0.000
Total Residuals	0.076	17	0.004		

**TABLE 6:** Analysis of Variance Results for ln C_max_ Between Two Formulations.

Source of variation	SS	DF	MS	F	P
Total Variation	2.156	37	0.058		
Inter-formulation Variation	0.005	1	0.005	0.433	0.519
Inter-period Variation	0.030	1	0.030	2.815	0.112
Inter-subject Variation	1.940	18	0.108	10.132	0.000
Total Residuals	0.181	17	0.011		

**TABLE 7:** Equivalence Analysis for ln AUC_0-84_ Using Two Sided One Sample *t*-Test.

	Numerical value (%)	P	Conclusion
Relative Bioavailability (F) of the Test Formulation	102.9%		
Equivalence Testing (Comparison to the Lower Bound)	12.174	0.000	Qualified
Equivalence Testing (Comparison to the Upper Bound)	9.370	0.000	Qualified
[1-2α]% Confidence Interval	99.3% ∼ 106.7%		Qualified

**TABLE 8:** Equivalence Analysis for ln AUC_0- ∞_ Using [1-2α]% Confidence Interval Method.

	Numerical value (%)	P	Conclusion
Relative Bioavailability (F) of the Test Formulation	103.4%		
Equivalence Testing (Comparison to the Lower Bound)	11.846	0.000	Qualified
Equivalence Testing (Comparison to the Upper Bound)	8.785	0.000	Qualified
[1-2α]% Confidence Interval	99.5%∼107.3%		Qualified

**TABLE 9:** Equivalence Analysis for ln C_max_ Using Two Way One Sided *t*-Test.

	Numerical value (%)	P	Conclusion
Relative Bioavailability (F) of the Test Formulation	102.2%		
Equivalence Testing (Comparison to the Lower Bound)	11.316	0.000	Qualified
Equivalence Testing (Comparison to the Upper Bound)	10.030	0.000	Qualified
[1-2α]% Confidence Interval	96.4%∼108.4%		Qualified

**TABLE 10:** Results of nonparametric test for t_max_ using paired wilcoxon method.

	Reference formulation	Test formulation	P	Conclusion
Mean ± SD	4.21 ± 1.55	4.32 ± 1.63	>0.05	Qualified
Max-Min	9.00–2.00	9.00–3.00		
Median	4	4		

### Tolerability

All 19 volunteer participants completed the trial. However, participant number 11 voluntarily withdrew shortly after the trial began due to personal reasons. Throughout the experimental period, there were no gastrointestinal adverse reactions such as nausea, stomach pain, or indigestion observed, nor were there any other symptoms like dizziness, tinnitus, headache, rash, or insomnia. This indicates that the medication was relatively safe to use, with no significant adverse reactions occurring.

## Discussion

Given the limited public disclosure of literature on rifapentine bioequivalence studies, and the existing reports often lacking in detailed methodology and comprehensive pharmacokinetic data for healthy individuals, this paper provides the first thorough report on the pharmacokinetics of a single oral dose of rifapentine in healthy subjects. This study aims to furnish robust support for future clinical pharmacokinetic research on rifapentine.

This study employed UPLC with an internal standard to determine rifapentine concentrations in the plasma of healthy subjects, assessing bioavailability and bioequivalence. Endogenous substances did not interfere with the analyses. The standard curve was linear from 0.05 to 20.00 μg/mL, with a quantification lower limit of 0.05 μg/mL. Recovery rates ranged from 102.60% to 109.55%, with both intra-day and inter-day relative standard deviations (RSDs) below 15%.

During sample preparation, multiple extraction methods were explored to optimize rifapentine recovery. Initial trials using protein precipitation with methanol, trichloroacetic acid, isopropyl acetate, and glacial acetic acid were quick but produced excessive chromatographic peaks, affecting analytical accuracy. Subsequent trials with liquid-liquid extraction using n-hexane, ethyl acetate, dichloromethane with methanol, and dichloromethane with isopropanol improved extraction efficiency but suffered from long processing times and significant peak tailing, likely due to complex interactions with plasma components. Attempts using chloroform also resulted in peak tailing and impurities.

Solid-phase extraction (SPE) with specific columns (Sep-Pak C18 and C8, as well as Bond Elut C18 and C8) was then employed, which increased selectivity but did not completely resolve issues with impurities and peak tailing and increased sample processing costs. Ultimately, direct protein precipitation with acetonitrile was chosen. This method, involving the simple addition of acetonitrile, efficiently precipitated plasma proteins, streamlined the process, significantly reduced chromatographic impurities, and enhanced reproducibility. Due to its simplicity, efficiency, and low equipment demands, acetonitrile precipitation was preferred, laying a strong foundation for further drug concentration and bioequivalence studies.

Compared to earlier rifapentine extraction methods (Winchester et al., 2015; Parsons et al., 2014; Riva et al., 1991; Panchagnula et al., 1999), this approach offers simplicity and speed in sample handling, short detection times, and low reagent consumption, achieving a low quantification limit without the need for expensive instruments, SPE columns, or extraction kits. This method aligns with NMPA and USFDA guidelines for biological sample analysis.

This study assessed rifapentine concentrations in plasma and calculated the pharmacokinetic parameters for the test formulation as follows: half-life (T_1/2_) was 15.845 ± 3.350 h, maximum concentration (C_max_) reached 12.401 ± 3.221 μg/mL, time to reach maximum concentration (T_max_) was 4.316 ± 1.635 h, area under the curve from 0 to 84 h (AUC_0-84_) was 369.471 ± 114.334 μg/mL•h, and area under the curve from 0 to infinity (AUC_0- ∞ _) was 384.456 ± 124.561 μg/mL•h. For the reference formulation, the parameters were: T_1/2_ at 15.422 ± 2.914 h, C_max_ at 12.093 ± 2.921 μg/mL, T_max_ at 4.211 ± 1.548 h, AUC_0-84_ at 357.867 ± 104.519 μg/mL•h, and AUC_0- ∞_ at 370.921 ± 114.284 μg/mL•h. Employing rifapentine capsules from Changzheng Pharmaceutical Co., Ltd. as the reference, the average relative bioavailability of rifapentine capsules produced by Everbright Pharma Co., Ltd. was calculated to be 103.4% ± 9.6%, which conforms to the bioequivalence standards set by the National Medical Products Administration (2009).

After variance analysis of the natural logarithms of AUC_0-84_ and AUC_0- ∞ _, no significant differences were found between the test and reference formulations of rifapentine capsules. The two-sided one-sample *t*-test revealed that the [1-2α]% confidence intervals for ln AUC_0-84_ and ln AUC_0- ∞_ were 99.3%–106.7% and 99.5%–107.3%, respectively, both within the 80%–125% range, indicating bioequivalence in terms of absorption. Similarly, variance analysis of ln C_max_ showed no significant differences between the two formulations. The [1-2α]% confidence interval for ln C_max_ was 96.4%–108.4%, within the 70%–143% range, demonstrating bioequivalence in peak concentration. T_max_, analyzed through non-parametric testing, also showed no significant differences between the formulations, indicating bioequivalence in the time to reach peak concentration.

The novelty of this study lies in the evaluation of the bioequivalence between a new generic rifapentine capsule (test formulation) and an approved reference formulation. While both products share the same active ingredient, differences in excipients and manufacturing processes could lead to variations in clinical outcomes. Therefore, demonstrating bioequivalence between the test and reference formulations is essential to ensure that patients receive consistent therapeutic effects when using either formulation.

Additionally, due to significant individual variability, careful consideration of individual differences is advised when administering the medication.

## Conclusion

In summary, this study established a rapid analytical method for detecting rifamycin-class drugs in human plasma, which can be utilized for pharmacokinetic research on rifamycin drugs. This method will aid in devising rational dosing regimens for future clinical trials of rifamycin-class medications and also provides valuable insights for optimizing clinical dosing strategies. Based on the experimental results described, both the test and reference formulations of rifapentine capsules were found to be bioequivalent in healthy, fasted Chinese male volunteers who were administered 600 mg orally in a crossover fashion. The results confirmed that the formulations meet the bioequivalence criteria set by the NMPA regulatory guidelines.

## Data Availability

The original contributions presented in the study are included in the article/Supplementary Material, further inquiries can be directed to the corresponding author.

## Supplementary material

SUPPLEMENTARY FIGURE S1 Daily average standard curves over a 3-day period. **(A)** Calibration curve for day 1. **(B)** Calibration curve for day 2. **(C)** Calibration curve for day 3.
