# Supplementary figures and images for: Comparative study on the bioavailability and bioequivalence of rifapentine capsules in humans
Source: Front Pharmacol. 2025 Jan 17;15:1463575. doi: 10.3389/fphar.2024.1463575 (PMC11782957; doi:10.3389/fphar.2024.1463575)

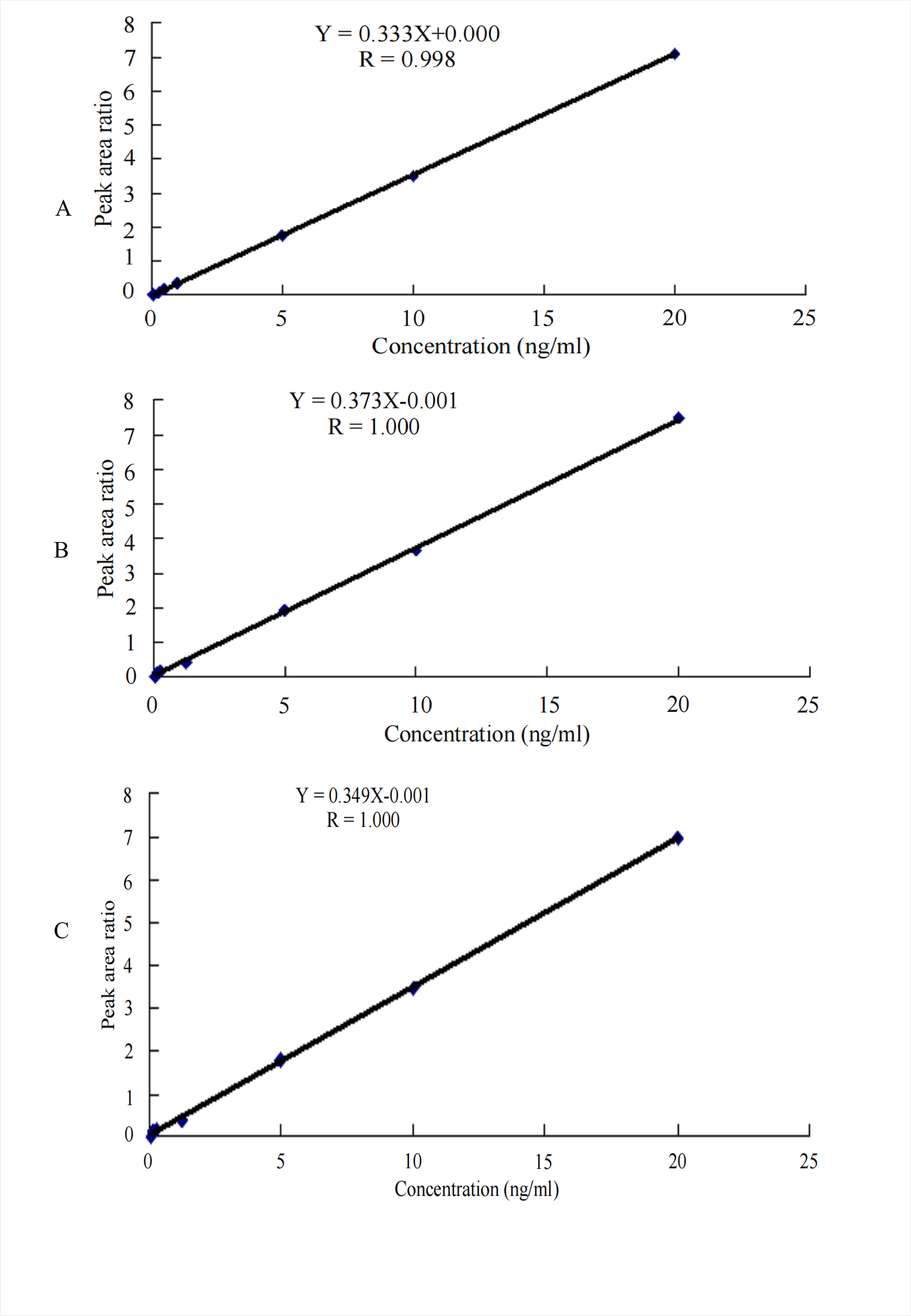

Supplement: Supplementary file 1 [file Image1.tif]
