# Supplementary material for: Comparative study on the bioavailability and bioequivalence of rifapentine capsules in humans
Source: Front Pharmacol. 2025 Jan 17;15:1463575. doi: 10.3389/fphar.2024.1463575 (PMC11782957; doi:10.3389/fphar.2024.1463575)
Supplement: Supplementary file 2 [file DataSheet1.docx]

**Table 1 Dosing Regimen.**

| Study Period | Period 1 | Washout Period | Period 2 |
| --- | --- | --- | --- |
| Group A | T | 7 days | R |
| Group B | R |  | T |

Note: T = Test Formulation, R = Reference Formulation

**Table 2 Day 1 Methodology Standard Curve Peak Area Ratios and Curve Equations.**

| Concentration (μg/ml)  Peak Area Ratios | 0.05 | 0.25 | 0.50 | 1.00 | 5.00 | 10.00 | 20.00 |
| --- | --- | --- | --- | --- | --- | --- | --- |
| F1 | 0.0178 | 0.0772 | 0.1621 | 0.3238 | 1.8726 | 3.6834 | 7.2585 |
| F2 | 0.0147 | 0.0702 | 0.1470 | 0.3013 | 1.5654 | 3.1572 | 6.6149 |
| F3 | 0.0183 | 0.0844 | 0.1810 | 0.3301 | 1.7242 | 3.6849 | 7.3971 |
| Mean | 0.0169 | 0.0773 | 0.1633 | 0.3184 | 1.7207 | 3.5085 | 7.0902 |
| Average Standard Curve Equation |  |  | Y =0.333X＋0.000 | |  |  |  |
| r |  |  | 0.998 |  |  |  |  |

**Table 3 Day 2 Methodology Standard Curve Peak Area Ratios and Curve Equations.**

| Concentration (μg/ml)  Peak Area Ratios | 0.05 | 0.25 | 0.50 | 1.00 | 5.00 | 10.00 | 20.00 |
| --- | --- | --- | --- | --- | --- | --- | --- |
| F1 | 0.0179 | 0.0998 | 0.1930 | 0.3742 | 1.9079 | 3.7204 | 7.5650 |
| F2 | 0.0141 | 0.1008 | 0.1904 | 0.3751 | 1.8959 | 3.7016 | 7.4774 |
| F3 | 0.0165 | 0.0889 | 0.1921 | 0.3700 | 1.8603 | 3.6374 | 7.3740 |
| Mean | 0.0161 | 0.0965 | 0.1918 | 0.3731 | 1.8880 | 3.6865 | 7.4721 |
| Average Standard Curve Equation |  |  | Y= 0.373X－0.001 | |  |  |  |
| r |  |  | 1.000 |  |  |  |  |

**Table 4 Day 3 Methodology Standard Curve Peak Area Ratios and Curve Equations.**

| Concentration (μg/ml)  Peak Area Ratios | 0.05 | 0.25 | 0.50 | 1.00 | 5.00 | 10.00 | 20.00 |
| --- | --- | --- | --- | --- | --- | --- | --- |
| F1 | 0.0190 | 0.0828 | 0.1814 | 0.3968 | 1.8872 | 3.7042 | 7.3792 |
| F2 | 0.0155 | 0.0805 | 0.1656 | 0.3560 | 1.7840 | 3.4892 | 7.0143 |
| F3 | 0.0152 | 0.0824 | 0.1732 | 0.3146 | 1.7491 | 3.2376 | 6.5606 |
| Mean | 0.0166 | 0.0819 | 0.1734 | 0.3558 | 1.8068 | 3.4770 | 6.9847 |
| Average Standard Curve Equation |  |  | Y =0.349X－0.001 | |  |  |  |
| r |  |  | 1.000 |  |  |  |  |

**Table 5 Precision and Accuracy of the Lower Limit of Quantification for Rifapentine in Plasma Samples Determined by HPLC-UV Method (n = 5).**

| Nominal Concentration (μg/ml) | Measured Concentration(μg/ml) | Accuracy (%) | Mean | SD | RSD (%) |
| --- | --- | --- | --- | --- | --- |
| 0.05 | 0.044 | 88.69 |  |  |  |
|  | 0.042 | 100.13 |  |  |  |
|  | 0.050 | 100.05 | 0.049 | 0.002 | 5.01 |
|  | 0.049 | 98.48 |  |  |  |
|  | 0.050 | 99.33 |  |  |  |

**Table 6 Intraday and Interday precision of Rifapentine plasma samples (n = 6).**

| Day | Concentration (μg/ml) | | | | | |
| --- | --- | --- | --- | --- | --- | --- |
|  | 0.1 | Accuracy (%) | 2.5 | Accuracy (%) | 16 | Accuracy (%) |
| 1 | 0.100 | 99.84 | 2.648 | 105.94 | 17.138 | 107.11 |
|  | 0.092 | 91.89 | 2.608 | 104.33 | 17.320 | 108.25 |
|  | 0.093 | 93.11 | 2.670 | 106.81 | 17.585 | 109.91 |
|  | 0.100 | 99.62 | 2.667 | 106.69 | 17.495 | 109.34 |
|  | 0.102 | 102.11 | 2.732 | 109.30 | 16.377 | 102.36 |
|  | 0.101 | 101.09 | 2.732 | 109.27 | 16.413 | 102.58 |
| Intraday SD | 0.004 |  | 0.049 |  | 0.534 |  |
| Mean | 0.098 |  | 2.676 |  | 17.055 |  |
| RSD(%) | 4.42 |  | 1.81 |  | 3.13 |  |
|  | | | | | |  |
| 2 | 0.106 | 106.25 | 2.466 | 98.64 | 16.466 | 102.91 |
|  | 0.093 | 92.93 | 2.442 | 97.69 | 16.068 | 100.43 |
|  | 0.112 | 111.53 | 2.257 | 90.27 | 15.994 | 99.96 |
|  | 0.096 | 95.82 | 2.383 | 95.34 | 16.839 | 105.24 |
|  | 0.094 | 94.46 | 2.381 | 95.26 | 16.382 | 102.39 |
|  | 0.091 | 90.74 | 2.353 | 94.14 | 16.145 | 100.90 |
| Intraday SD | 0.008 |  | 0.074 |  | 0.314 |  |
| Mean | 0.099 |  | 2.381 |  | 16.316 |  |
| RSD(%) | 8.42 |  | 3.10 |  | 1.93 |  |
|  | | | | | |  |
| 3 | 0.103 | 103.05 | 2.616 | 104.63 | 17.765 | 111.03 |
|  | 0.096 | 96.21 | 2.668 | 106.71 | 17.403 | 108.77 |
|  | 0.092 | 91.71 | 2.670 | 106.78 | 17.470 | 109.19 |
|  | 0.086 | 86.31 | 2.604 | 104.16 | 16.594 | 103.71 |
|  | 0.093 | 93.12 | 2.516 | 100.64 | 15.443 | 96.52 |
|  | 0.095 | 94.71 | 2.556 | 102.22 | 17.137 | 107.10 |
| Intraday SD | 0.006 |  | 0.061 |  | 0.845 |  |
| Mean | 0.094 |  | 2.605 |  | 16.968 |  |
| RSD(%) | 5.86 |  | 2.34 |  | 4.98 |  |
|  | | | | | |  |
| Interday SD | 0.006 |  | 0.142 |  | 0.662 |  |
| Mean | 0.097 |  | 2.554 |  | 16.780 |  |
| RSD(%) | 6.42 |  | 5.56 |  | 3.94 |  |

**Table 7-1 Extraction Recovery Rate of Rifapentine in Plasma(n = 3).**

| Concentration (μg/ml) | | | |
| --- | --- | --- | --- |
|  | 0.10 | 2.50 | 16.00 |
| Recovery Rate (%) | 103.56 | 108.98 | 102.60 |
|  | 103.65 | 109.55 | 106.98 |
|  | 102.86 | 107.15 | 108.29 |
| SD | 0.430 | 1.253 | 2.980 |
| Mean | 103.358 | 108.559 | 105.958 |
| RSD (%) | 0.42 | 1.154 | 2.812 |

**Table 7-2 Extraction Recovery Rate of Rifampin in Plasma (n = 3).**

| Concentration (μg/ml) | | | |
| --- | --- | --- | --- |
|  | 62.2 | 62.2 | 62.2 |
| Recovery Rate (%) | 108.28 | 112.04 | 99.19 |
|  | 108.42 | 110.00 | 105.20 |
|  | 108.46 | 108.39 | 101.04 |
| SD | 0.097 | 1.827 | 3.079 |
| Mean | 108.386 | 110.143 | 101.810 |
| RSD (%) | 0.09 | 1.659 | 3.025 |

**Table 8-1 Stability investigation of Rifapentine in Plasma Samples (n = 3).**

| Storage Time (h) | 0.1 μg/ml | Accuracy (%) | 16 μg/ml | Accuracy (%) |
| --- | --- | --- | --- | --- |
| 0h | 0.100 | 99.84 | 17.138 | 107.11 |
|  | 0.092 | 91.89 | 17.320 | 108.25 |
|  | 0.095 | 94.90 | 16.982 | 106.14 |
| SD | 0.004 |  | 0.169 |  |
| Mean | 0.096 |  | 17.147 |  |
| RSD(%) | 4.20 |  | 0.99 |  |
| 24h | 0.106 | 106.282 | 16.568 | 103.550 |
|  | 0.101 | 101.230 | 14.804 | 92.524 |
|  | 0.093 | 92.626 | 14.963 | 93.520 |
| SD | 0.006 |  | 1.123 |  |
| Mean | 0.098 |  | 16.296 |  |
| RSD(%) | 5.75 |  | 6.89 |  |
| 36h | 0.083 | 82.73 | 13.855 | 86.60 |
|  | 0.094 | 93.78 | 14.431 | 90.19 |
|  | 0.088 | 88.29 | 14.419 | 90.12 |
| SD | 0.006 |  | 1.612 |  |
| Mean | 0.092 |  | 15.691 |  |
| RSD(%) | 6.40 |  | 10.27 |  |
| Plasma Thawing for 8 Hours | 0.094 | 94.12 | 15.621 | 97.63 |
|  | 0.107 | 107.04 | 15.416 | 96.35 |
|  | 0.104 | 103.80 | 16.043 | 100.27 |
| SD | 0.006 |  | 0.828 |  |
| Mean | 0.099 |  | 16.420 |  |
| RSD(%) | 6.06 |  | 5.04 |  |
| Frozen for 15 Days | 0.106 | 105.56 | 16.402 | 102.51 |
|  | 0.103 | 103.18 | 17.165 | 107.28 |
|  | 0.091 | 91.25 | 16.089 | 100.56 |
| SD | 0.006 |  | 0.490 |  |
| Mean | 0.098 |  | 16.849 |  |
| RSD(%) | 6.13 |  | 2.91 |  |
| Frozen for 30 Days | 0.108 | 107.69 | 17.708 | 110.68 |
|  | 0.100 | 100.10 | 17.836 | 111.48 |
|  | 0.104 | 104.10 | 18.228 | 113.93 |
| SD | 0.006 |  | 0.471 |  |
| Mean | 0.100 |  | 17.536 |  |
| RSD(%) | 5.80 |  | 2.69 |  |

**Table 8-2 Stability investigation of Rifapentine in Plasma Samples after three freeze-thaw cycles (n = 3).**

| Freeze-thaw cycles (times) | 0.1 μg/ml | Accuracy (%) | 16μg/ml | Accuracy (%) |
| --- | --- | --- | --- | --- |
| 1 | 0.091 | 90.51 | 15.831 | 98.95 |
|  | 0.088 | 87.72 | 15.843 | 99.02 |
|  | 0.091 | 91.28 | 15.481 | 96.75 |
| SD | 0.004 |  | 0.800 |  |
| Mean | 0.093 |  | 16.432 |  |
| RSD(%) | 4.53 |  | 4.87 |  |
| 2 | 0.096 | 96.37 | 17.437 | 108.98 |
|  | 0.095 | 95.06 | 17.255 | 107.85 |
|  | 0.094 | 93.74 | 17.210 | 107.56 |
| SD | 0.003 |  | 0.156 |  |
| Mean | 0.095 |  | 17.224 |  |
| RSD(%) | 2.82 |  | 0.91 |  |
| 3 | 0.092 | 92.00 | 18.211 | 113.82 |
|  | 0.089 | 89.13 | 17.528 | 109.55 |
|  | 0.090 | 89.83 | 17.801 | 111.25 |
| SD | 0.004 |  | 0.454 |  |
| Mean | 0.093 |  | 17.497 |  |
| RSD(%) | 4.24 |  | 2.59 |  |

**Table 9 Stability Assessment of Rifapentine Standard (n = 3).**

| Storage Time | Peak Area Ratio (Rifapentine/Rifampin) | |
| --- | --- | --- |
|  | 0.1 μg/ml | 16 μg/ml |
| 0h | 0.032 | 5.739 |
|  | 0.029 | 5.856 |
|  | 0.032 | 5.704 |
| SD | 0.001 | 0.080 |
| Mean | 0.031 | 5.766 |
| RSD (%) | 4.29 | 1.39 |
| 8h (Room Temperature Storage) | 0.031 | 5.595 |
|  | 0.031 | 6.061 |
|  | 0.032 | 5.682 |
| SD | 0.001 | 0.165 |
| Mean | 0.031 | 5.773 |
| RSD (%) | 2.99 | 2.86 |
| Freshly Prepared | 0.029 | 5.751 |
|  | 0.028 | 5.707 |
|  | 0.027 | 5.683 |
| SD | 0.001 | 0.035 |
| Mean | 0.028 | 5.714 |
| RSD(%) | 4.35 | 0.61 |
| 30 days (Frozen Storage) | 0.028 | 5.575 |
|  | 0.028 | 5.661 |
|  | 0.029 | 5.469 |
| SD | 0.001 | 0.102 |
| Mean | 0.028 | 5.641 |
| RSD(%) | 3.08 | 1.81 |

**Table 10-1 Peak Area Ratios and Equations of Concurrent Standard Curves for Rifapentine**

| Concentration (μg/ml) | F1 | F2 | F3 |
| --- | --- | --- | --- |
| 0.05 | 0.017 | 0.017 | 0.020 |
| 0.25 | 0.085 | 0.085 | 0.099 |
| 0.5 | 0.178 | 0.182 | 0.191 |
| 1 | 0.340 | 0.356 | 0.370 |
| 5 | 1.624 | 1.912 | 1.769 |
| 10 | 3.211 | 3.775 | 3.297 |
| 20 | 6.660 | 7.569 | 7.379 |
| Curve Equation | Y=0.335X+0.000 | Y=0.368X－0.001 | Y=0.361X+0.002 |
| R | 0.999 | 0.999 | 0.998 |

**Table 10-2 Peak Area Ratios and Equations of Concurrent Standard Curves for Rifapentine.**

| Concentration (μg/ml) | F4 | F5 | F6 |
| --- | --- | --- | --- |
| 0.05 | 0.017 | 0.019 | 0.020 |
| 0.25 | 0.090 | 0.092 | 0.095 |
| 0.5 | 0.182 | 0.202 | 0.192 |
| 1 | 0.372 | 0.363 | 0.388 |
| 5 | 1.845 | 1.822 | 1.836 |
| 10 | 3.656 | 3.514 | 3.419 |
| 20 | 7.837 | 7.697 | 7.179 |
| Curve Equation | Y=0.372X－0.001 | Y=0.370+0.000 | Y=0.366X+0.001 |
| R | 1.000 | 0.999 | 0.999 |

**Table 10-3 Peak Area Ratios and Equations of Concurrent Standard Curves for Rifapentine.**

| Concentration (μg/ml) | F7 | F8 |  |
| --- | --- | --- | --- |
| 0.05 | 0.019 | 0.017 |  |
| 0.25 | 0.091 | 0.091 |  |
| 0.5 | 0.184 | 0.144 |  |
| 1 | 0.385 | 0.328 |  |
| 5 | 1.678 | 1.645 |  |
| 10 | 3.268 | 3.103 |  |
| 20 | 6.382 | 7.247 |  |
| Curve Equation | Y=0.343X+0.002 | Y=0.325X+0.001 |  |
| R | 0.998 | 0.996 |  |

**Table 11 Measured Concentration, Mean, and Standard Deviation of Rifapentine Quality Control Samples.**

| Sample batch | Concentration (μg/ml) | | | | | |
| --- | --- | --- | --- | --- | --- | --- |
|  | 0.1 Accuracy (%) | | 2.5 Accuracy (%) | | 16 Accuracy (%) | |
| 1  2  3  4  5  6  7  8  9  10  11  12  13  14  15  16  17  18  19  20  21  22  23  24  25  26 | 0.101 | 100.68 | 2.550 | 102.01 | 16.500 | 103.12 |
|  | 0.095 | 95.48 | 2.529 | 101.17 | 17.443 | 109.02 |
|  | 0.104 | 103.64 | 2.528 | 101.11 | 18.114 | 113.21 |
|  | 0.099 | 99.42 | 2.628 | 105.12 | 17.427 | 108.92 |
|  | 0.104 | 103.98 | 2.217 | 88.70 | 17.066 | 106.66 |
|  | 0.101 | 101.21 | 2.522 | 100.88 | 15.365 | 96.03 |
|  | 0.095 | 95.07 | 2.581 | 103.24 | 16.369 | 102.30 |
|  | 0.103 | 103.24 | 2.420 | 96.82 | 16.107 | 100.67 |
|  | 0.101 | 100.56 | 2.290 | 91.60 | 15.692 | 98.07 |
|  | 0.091 | 91.36 | 2.548 | 101.91 | 16.868 | 105.42 |
|  | 0.093 | 93.05 | 2.548 | 101.91 | 16.186 | 101.16 |
|  | 0.093 | 92.58 | 2.369 | 94.76 | 15.330 | 95.81 |
|  | 0.094 | 93.61 | 2.216 | 88.65 | 14.311 | 89.44 |
|  | 0.092 | 91.81 | 2.178 | 87.11 | 13.931 | 87.07 |
|  | 0.106 | 106.37 | 2.552 | 102.09 | 17.341 | 108.38 |
|  | 0.103 | 102.98 | 2.430 | 97.21 | 15.916 | 99.47 |
|  | 0.096 | 96.46 | 2.513 | 100.50 | 16.591 | 103.70 |
|  | 0.099 | 99.40 | 2.359 | 94.35 | 16.802 | 105.01 |
|  | 0.098 | 97.951 | 2.295 | 91.816 | 15.426 | 96.41 |
|  | 0.105 | 105.293 | 2.142 | 85.692 | 15.360 | 96.00 |
|  | 0.105 | 104.982 | 2.565 | 102.590 | 15.438 | 96.49 |
|  | 0.092 | 91.775 | 2.544 | 101.751 | 16.419 | 102.62 |
|  | 0.101 | 100.66 | 2.702 | 108.07 | 16.628 | 103.93 |
|  | 0.098 | 98.27 | 2.631 | 105.26 | 16.637 | 103.98 |
|  | 0.099 | 99.43 | 2.616 | 104.62 | 17.323 | 108.27 |
|  | 0.109 | 108.94 | 2.585 | 103.41 | 15.842 | 99.01 |
| SD | 0.005 |  | 0.156 |  | 0.980 |  |
| Mean | 0.099 |  | 2.464 |  | 16.247 |  |
| RSD(%) | 5.04 |  | 6.35 |  | 6.03 |  |

**Table 12 Plasma Concentration-Time Data of Rifapentine (μg/ml) in 19 Subjects Following Oral Administration of 0.6g Reference Rifapentine Capsules.**

| Subject Code |  |  |  |  |  |  | Time (h) |  |  |  |  |  |  |  |
| --- | --- | --- | --- | --- | --- | --- | --- | --- | --- | --- | --- | --- | --- | --- |
|  | 0 | 1 | 2 | 3 | 4 | 5 | 7 | 9 | 12 | 24 | 36 | 48 | 72 | 84 |
| 1 | 0 | 0.171 | 4.431 | 7.409 | 8.995 | 8.160 | 8.851 | 8.987 | 8.926 | 6.077 | 4.962 | 1.969 | 0.624 | 0.399 |
| 2 | 0 | 0.172 | 4.224 | 7.282 | 9.768 | 9.626 | 11.815 | 10.426 | 9.266 | 6.030 | 3.420 | 2.008 | 0.492 | 0.136 |
| 3 | 0 | 0.752 | 8.872 | 8.670 | 11.679 | 11.286 | 10.672 | 10.438 | 9.553 | 8.416 | 7.558 | 4.799 | 2.039 | 1.255 |
| 4 | 0 | 0.082 | 3.095 | 5.163 | 12.861 | 15.458 | 13.235 | 14.177 | 12.364 | 7.536 | 6.816 | 3.427 | 1.110 | 0.584 |
| 5 | 0 | 1.392 | 5.989 | 8.135 | 12.671 | 12.032 | 11.241 | 11.903 | 11.187 | 8.853 | 6.550 | 4.401 | 1.700 | 0.900 |
| 6 | 0 | 0.177 | 4.490 | 7.348 | 10.137 | 10.357 | 10.116 | 8.768 | 9.423 | 5.542 | 3.683 | 2.089 | 0.540 | 0.220 |
| 7 | 0 | 0.126 | 4.493 | 9.468 | 9.038 | 9.199 | 8.784 | 6.965 | 8.539 | 5.701 | 4.739 | 1.596 | 0.297 | 0.176 |
| 8 | 0 | 6.530 | 10.435 | 9.539 | 8.958 | 7.305 | 7.125 | 6.818 | 5.663 | 4.129 | 3.482 | 1.548 | 0.730 | 0.441 |
| 9 | 0 | 1.166 | 6.009 | 10.203 | 12.816 | 10.279 | 11.032 | 10.132 | 8.404 | 6.655 | 5.358 | 2.428 | 0.880 | 0.587 |
| 10 | 0 | 1.577 | 5.898 | 7.491 | 9.972 | 8.165 | 7.656 | 6.306 | 5.871 | 4.052 | 2.051 | 1.786 | 0.870 | 0.190 |
| 12 | 0 | 0.353 | 2.806 | 8.668 | 17.158 | 14.542 | 13.268 | 13.094 | 11.847 | 8.489 | 4.936 | 3.127 | 1.000 | 0.579 |
| 13 | 0 | 0.167 | 0.728 | 3.048 | 7.692 | 3.823 | 3.205 | 2.908 | 3.104 | 4.152 | 1.606 | 1.083 | 0.830 | 0.278 |
| 14 | 0 | 0.615 | 10.690 | 12.205 | 15.112 | 13.980 | 14.595 | 13.267 | 12.191 | 8.151 | 6.743 | 3.539 | 1.737 | 0.886 |
| 15 | 0 | 3.436 | 13.965 | 17.455 | 17.497 | 12.799 | 13.013 | 12.275 | 12.581 | 9.074 | 6.893 | 5.390 | 2.041 | 0.593 |
| 16 | 0 | 0.176 | 4.110 | 9.120 | 9.116 | 7.744 | 8.815 | 8.416 | 7.636 | 5.020 | 2.734 | 1.543 | 0.299 | 0.146 |
| 17 | 0 | 0.491 | 2.655 | 8.925 | 10.672 | 9.520 | 9.184 | 8.411 | 7.824 | 5.332 | 4.143 | 2.143 | 0.547 | 0.288 |
| 18 | 0 | 5.338 | 12.710 | 15.325 | 12.531 | 12.416 | 14.364 | 10.055 | 11.069 | 6.706 | 4.488 | 2.889 | 1.046 | 0.391 |
| 19 | 0 | 0.286 | 5.631 | 6.855 | 8.860 | 8.458 | 9.025 | 9.572 | 8.114 | 6.077 | 3.597 | 2.339 | 1.001 | 0.444 |
| 20 | 0 | 0.112 | 3.525 | 13.953 | 12.987 | 11.951 | 10.936 | 10.822 | 9.378 | 6.859 | 5.063 | 1.990 | 0.383 | 0.200 |
| Mean | 0.000 | 1.217 | 6.040 | 9.277 | 11.501 | 10.374 | 10.365 | 9.670 | 9.102 | 6.466 | 4.675 | 2.636 | 0.956 | 0.458 |
| SD | 0.000 | 1.858 | 3.621 | 3.440 | 2.820 | 2.865 | 2.797 | 2.754 | 2.498 | 1.609 | 1.698 | 1.194 | 0.552 | 0.300 |
| RSD(%) |  | 152.7 | 59.9 | 37.1 | 24.5 | 27.6 | 27.0 | 28.5 | 27.4 | 24.9 | 36.3 | 45.3 | 57.8 | 65.6 |

Note:‘nd’ indicates not detected or below the limit of quantification.

**Table 13 Plasma Concentration-Time Data of Rifapentine (μg/ml) in 19 Subjects Following Oral Administration of 0.6g Test Rifapentine Capsules.**

| Subject Code |  |  |  |  |  |  | Time (h) |  |  |  |  |  |  |  |
| --- | --- | --- | --- | --- | --- | --- | --- | --- | --- | --- | --- | --- | --- | --- |
|  | 0 | 1 | 2 | 3 | 4 | 5 | 7 | 9 | 12 | 24 | 36 | 48 | 72 | 84 |
| 1 | 0 | 0.216 | 8.964 | 10.144 | 9.683 | 8.654 | 9.388 | 9.138 | 7.825 | 5.377 | 2.803 | 1.649 | 1.252 | 0.338 |
| 2 | 0 | 0.476 | 2.806 | 9.862 | 11.232 | 9.118 | 8.971 | 8.547 | 7.923 | 5.223 | 3.747 | 2.154 | 0.608 | 0.309 |
| 3 | 0 | 0.148 | 0.909 | 1.590 | 11.391 | 14.197 | 14.387 | 18.080 | 12.812 | 11.015 | 7.916 | 4.133 | 3.087 | 0.935 |
| 4 | 0 | 0.127 | 9.874 | 12.428 | 11.650 | 11.846 | 12.330 | 11.594 | 10.988 | 10.358 | 6.410 | 4.999 | 1.582 | 0.758 |
| 5 | 0 | 0.132 | 6.772 | 9.125 | 12.956 | 10.281 | 8.087 | 9.302 | 5.990 | 9.344 | 7.034 | 4.369 | 3.445 | 0.985 |
| 6 | 0 | 0.551 | 2.772 | 10.311 | 11.864 | 9.895 | 9.568 | 9.209 | 8.150 | 5.445 | 3.436 | 1.939 | 0.509 | 0.220 |
| 7 | 0 | 0.147 | 8.332 | 9.680 | 9.584 | 6.778 | 8.952 | 8.603 | 7.465 | 6.083 | 2.571 | 1.999 | 0.547 | 0.371 |
| 8 | 0 | 0.366 | 3.689 | 12.328 | 11.746 | 9.592 | 8.414 | 7.808 | 7.134 | 6.315 | 2.984 | 1.309 | 0.559 | 0.375 |
| 9 | 0 | 0.403 | 2.610 | 7.270 | 13.018 | 12.337 | 9.017 | 8.120 | 7.790 | 6.501 | 4.813 | 1.874 | 0.447 | 0.352 |
| 10 | 0 | 2.852 | 6.357 | 8.527 | 9.162 | 8.326 | 8.274 | 7.569 | 6.685 | 4.750 | 2.620 | 1.527 | 0.172 | 0.122 |
| 12 | 0 | 0.340 | 5.313 | 10.404 | 12.642 | 10.580 | 11.226 | 11.045 | 9.529 | 10.396 | 5.544 | 3.756 | 0.949 | 0.406 |
| 13 | 0 | 1.517 | 1.925 | 2.633 | 7.544 | 6.663 | 4.392 | 3.592 | 3.091 | 3.448 | 2.296 | 1.650 | 0.551 | 0.158 |
| 14 | 0 | 1.972 | 9.142 | 12.215 | 15.499 | 13.788 | 14.421 | 13.014 | 13.293 | 7.996 | 5.173 | 2.264 | 1.720 | 0.765 |
| 15 | 0 | 7.344 | 10.517 | 19.965 | 14.539 | 12.607 | 12.456 | 11.169 | 10.308 | 9.206 | 8.541 | 5.695 | 2.689 | 0.814 |
| 16 | 0 | 0.134 | 4.084 | 8.935 | 8.740 | 7.503 | 8.644 | 8.026 | 6.833 | 5.823 | 5.243 | 2.057 | 0.603 | 0.382 |
| 17 | 0 | 0.154 | 4.270 | 7.448 | 9.915 | 8.638 | 10.347 | 9.270 | 9.355 | 5.902 | 3.795 | 1.917 | 0.451 | 0.129 |
| 18 | 0 | 0.164 | 4.620 | 14.025 | 16.351 | 15.449 | 14.117 | 13.258 | 12.350 | 8.545 | 5.125 | 3.137 | 1.712 | 1.103 |
| 19 | 0 | 0.265 | 3.544 | 5.840 | 7.475 | 7.540 | 10.002 | 7.947 | 7.053 | 6.308 | 4.020 | 2.320 | 1.185 | 0.272 |
| 20 | 0 | 0.465 | 2.280 | 6.024 | 12.388 | 13.439 | 11.798 | 10.341 | 9.797 | 7.012 | 6.380 | 4.155 | 0.811 | 0.368 |
| Mean | 0.000 | 0.935 | 5.199 | 9.408 | 11.441 | 10.381 | 10.252 | 9.770 | 8.651 | 7.108 | 4.760 | 2.784 | 1.204 | 0.482 |
| SD | 0.000 | 1.717 | 2.953 | 4.093 | 2.471 | 2.667 | 2.549 | 2.972 | 2.549 | 2.147 | 1.865 | 1.319 | 0.953 | 0.307 |
| RSD(%) |  | 183.5 | 56.8 | 43.5 | 21.6 | 25.7 | 24.9 | 30.4 | 29.5 | 30.2 | 39.2 | 47.4 | 79.1 | 63.8 |

Note:‘nd’ indicates not detected or below the limit of quantification.

**Table 14 Pre-Trial Physical Examination Data of Subjects**

| Subject Code | Gender | Age | Height | Weight | Temp | Heart Rate | Breath | Systolic Pressure | Diastolic Pressure | WBC | RBC | Hb | PLT | AST | ALT | BUN | Cr | Urinalysis | Electrocardiogram |
| --- | --- | --- | --- | --- | --- | --- | --- | --- | --- | --- | --- | --- | --- | --- | --- | --- | --- | --- | --- |
|  | M/F | Year | cm | kg | ℃ | 次/min | 次/min | mmHg | mmHg | 10^9^/L | 10^12^/L | g/L | 10^9^/L | U/L | U/L | mmol/L | μmol/L |  |  |
| 1 | M | 21 | 178 | 70 | 36.0 | 70 | 18 | 110 | 70 | 5.3 | 5.2 | 157 | 218 | 10 | 19 | 4.74 | 88 | Normal | Normal |
| 2 | M | 21 | 182 | 78 | 36.4 | 68 | 18 | 110 | 70 | 6.0 | 5.03 | 154 | 287 | 20 | 16 | 4.93 | 90 | Normal | Normal |
| 3 | M | 27 | 178 | 76 | 36.6 | 70 | 18 | 100 | 65 | 5.6 | 5.17 | 154 | 211 | 29 | 39 | 6.61 | 81 | Normal | Normal |
| 4 | M | 23 | 180 | 76 | 36.3 | 68 | 18 | 110 | 70 | 7.5 | 4.93 | 159 | 270 | 19 | 13 | 5.09 | 78 | Normal | Normal |
| 5 | M | 26 | 183 | 72 | 36.2 | 76 | 18 | 120 | 80 | 5.1 | 4.71 | 148 | 209 | 22 | 9 | 4.02 | 68 | Normal | Normal |
| 6 | M | 20 | 180 | 75 | 36.2 | 76 | 18 | 100 | 70 | 4.0 | 5.26 | 155 | 162 | 15 | 14 | 4.11 | 68 | Normal | Normal |
| 7 | M | 20 | 170 | 68 | 36.0 | 78 | 18 | 115 | 75 | 6.8 | 5.12 | 157 | 228 | 20 | 21 | 5.35 | 68 | Normal | Normal |
| 8 | M | 23 | 170 | 68 | 36.3 | 68 | 18 | 105 | 75 | 6.6 | 5.13 | 156 | 227 | 14 | 21 | 5.87 | 97 | Normal | Normal |
| 9 | M | 22 | 172 | 71 | 36.2 | 78 | 18 | 110 | 70 | 5.7 | 5.13 | 143 | 397 | 37 | 40 | 5.57 | 96 | Normal | Normal |
| 10 | M | 22 | 171 | 65 | 36.0 | 76 | 18 | 110 | 75 | 4.7 | 4.91 | 149 | 211 | 18 | 18 | 6.13 | 58 | Normal | Normal |
| 11 | M | 24 | 178 | 65 | 36.0 | 80 | 20 | 115 | 75 | 7.4 | 4.64 | 153 | 172 | 16 | 9 | 5.74 | 70 | Normal | Normal |
| 12 | M | 28 | 171 | 65 | 36.2 | 76 | 18 | 105 | 75 | 8.0 | 4.56 | 145 | 185 | 23 | 31 | 3.09 | 65 | Normal | Normal |
| 13 | M | 23 | 173 | 63 | 36.1 | 76 | 18 | 105 | 75 | 6.3 | 4.94 | 146 | 170 | 36 | 37 | 5.03 | 76 | Normal | Normal |
| 14 | M | 26 | 169 | 62 | 36.3 | 74 | 18 | 115 | 85 | 6.8 | 5.77 | 158 | 159 | 20 | 20 | 3.04 | 66 | Normal | Normal |
| 15 | M | 28 | 175 | 60 | 36.2 | 78 | 18 | 110 | 70 | 6.1 | 4.49 | 143 | 227 | 22 | 16 | 3.87 | 62 | Normal | Normal |
| 16 | M | 21 | 170 | 60 | 36.5 | 70 | 18 | 120 | 80 | 6.9 | 5.0 | 152 | 243 | 17 | 20 | 5.03 | 56 | Normal | Normal |
| 17 | M | 22 | 170 | 60 | 36.2 | 80 | 20 | 120 | 75 | 4.5 | 4.59 | 145 | 221 | 17 | 14 | 5.07 | 83 | Normal | Normal |
| 18 | M | 20 | 165 | 58 | 36.5 | 68 | 18 | 110 | 70 | 6.7 | 5.03 | 154 | 207 | 16 | 11 | 5.59 | 70 | Normal | Normal |
| 19 | M | 28 | 165 | 55 | 36.2 | 78 | 18 | 110 | 70 | 8.5 | 4.88 | 156 | 195 | 21 | 33 | 3 | 68 | Normal | Normal |
| 20 | M | 23 | 160 | 50 | 36.2 | 60 | 18 | 100 | 70 | 5.7 | 5.0 | 146 | 164 | 26 | 38 | 4.64 | 70 | Normal | Normal |
| MEAN |  | 23.40 | 173.00 | 65.85 | 36.23 | 73.40 | 18.20 | 110.00 | 73.25 | 6.21 | 4.97 | 151.50 | 218.15 | 20.90 | 21.95 | 4.83 | 73.90 |  |  |
| SD |  | 2.80 | 6.14 | 7.56 | 0.17 | 5.32 | 0.62 | 6.28 | 4.67 | 1.17 | 0.29 | 5.34 | 54.42 | 6.80 | 10.44 | 1.03 | 11.87 |  |  |
| Normal value |  |  |  |  |  |  |  |  |  | 3.97－9.15 | 4.09  －  5.74 | 131  －  172 | 85  －  303 | 8  －  40 | 5  －  40 | 2.9  －  8.2 | 44  －  80 |  |  |
